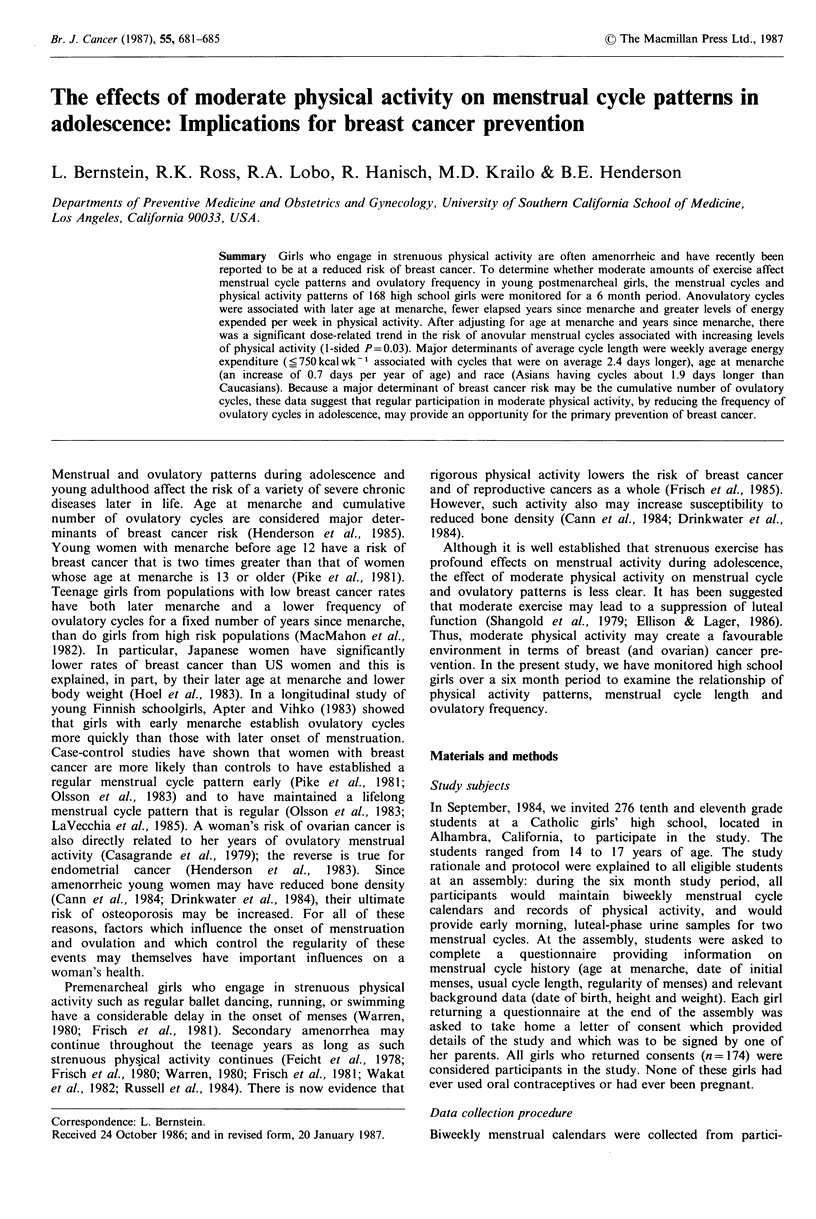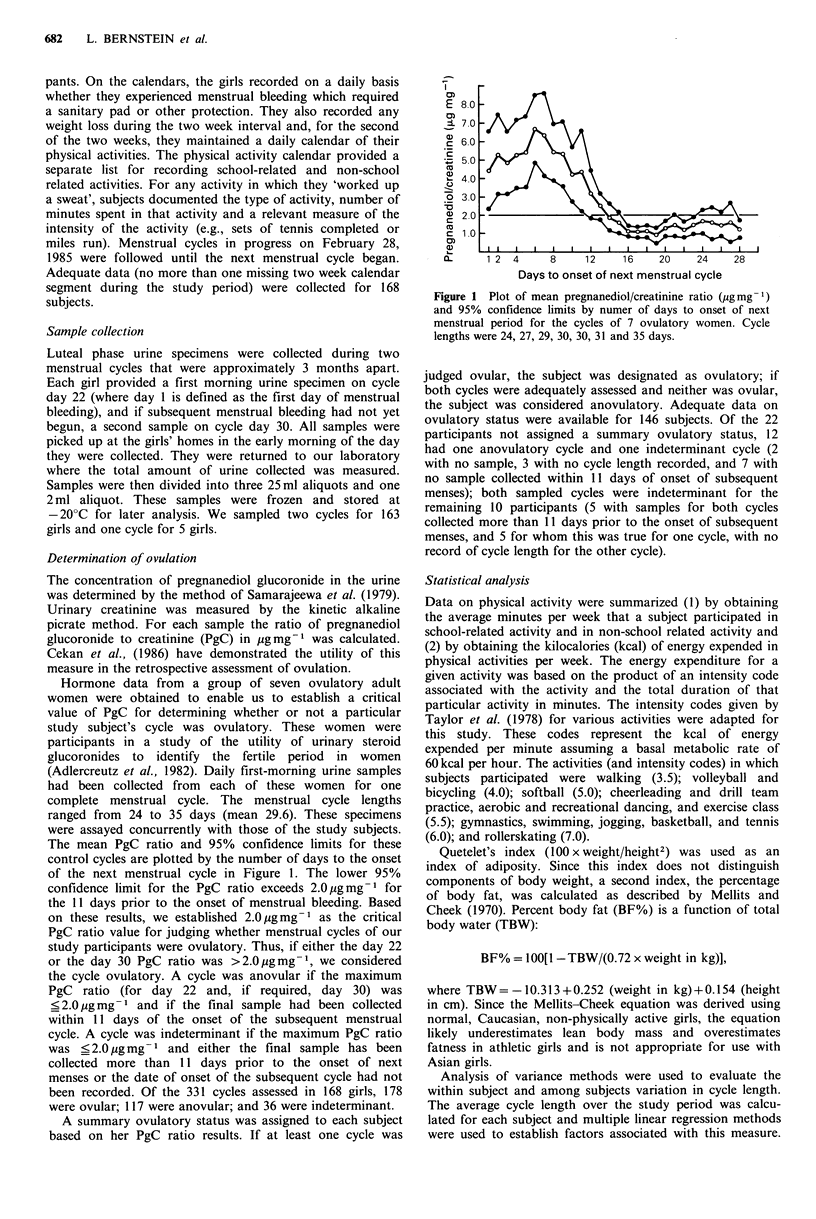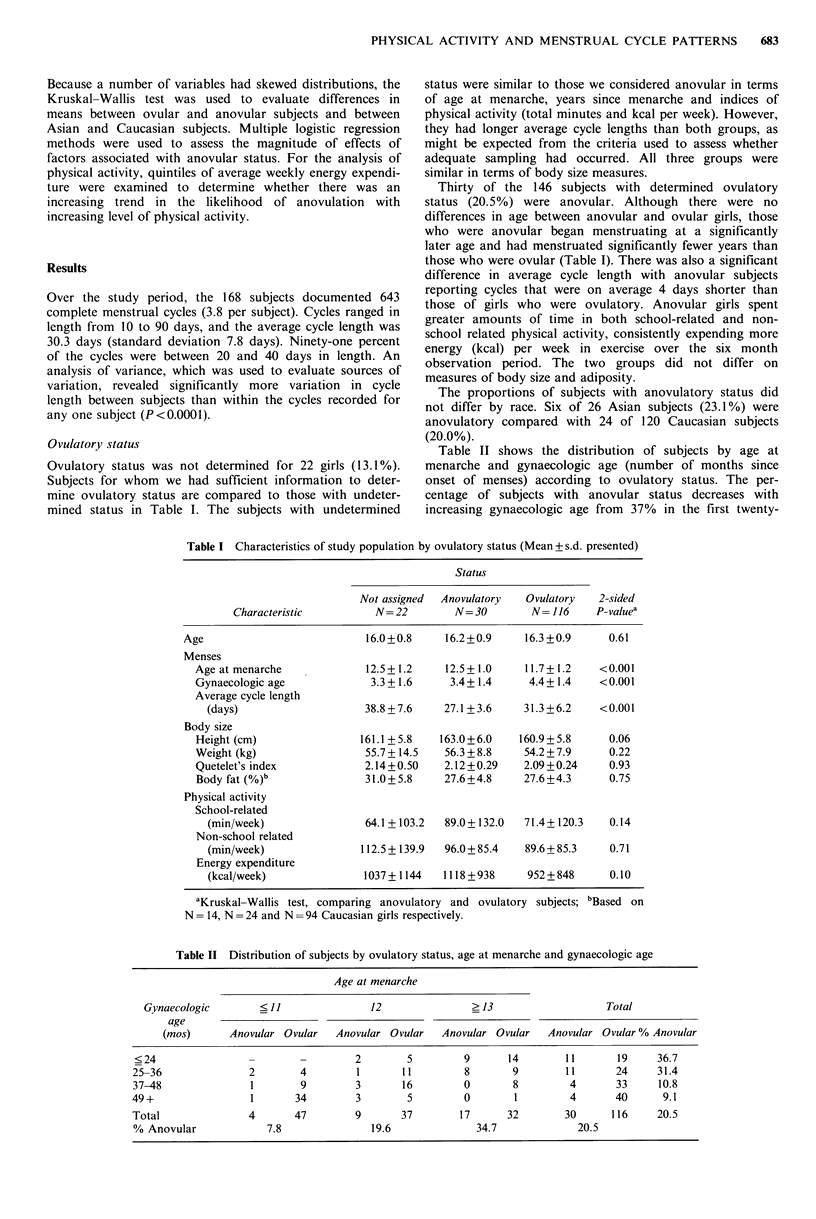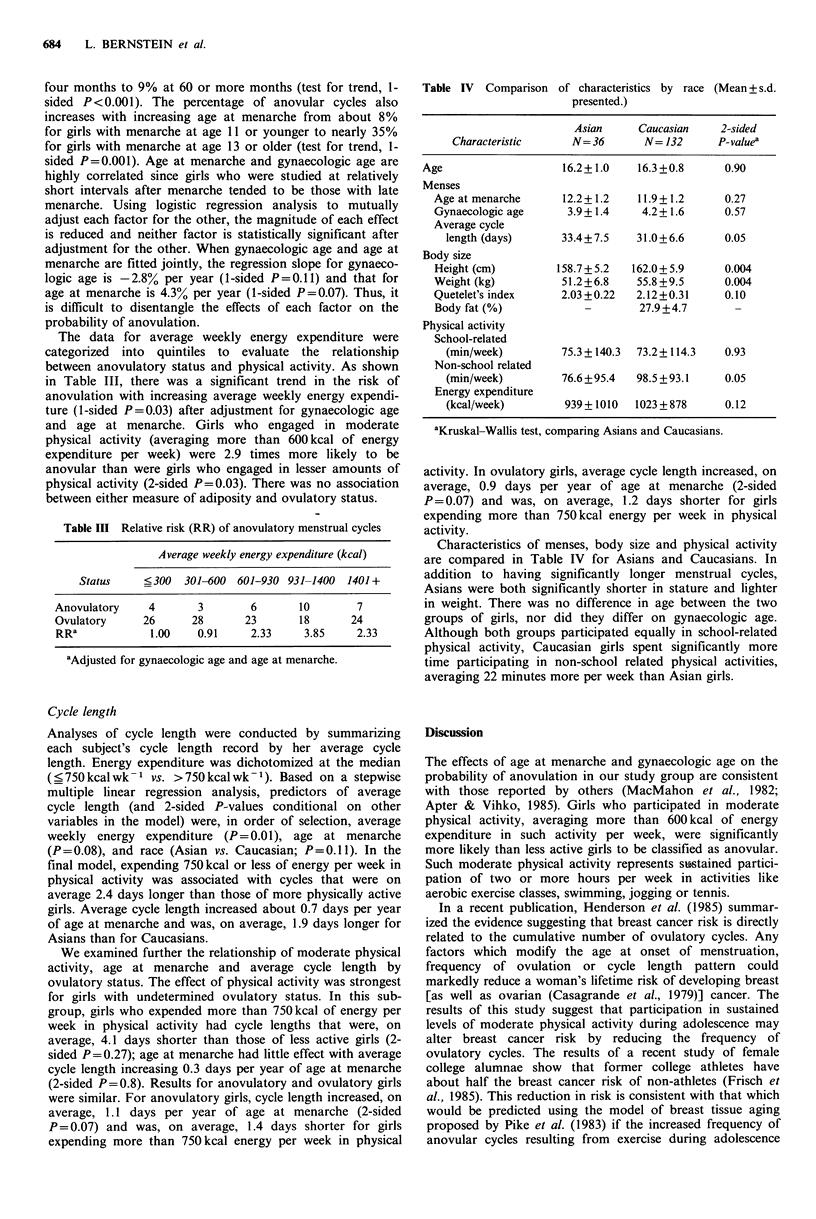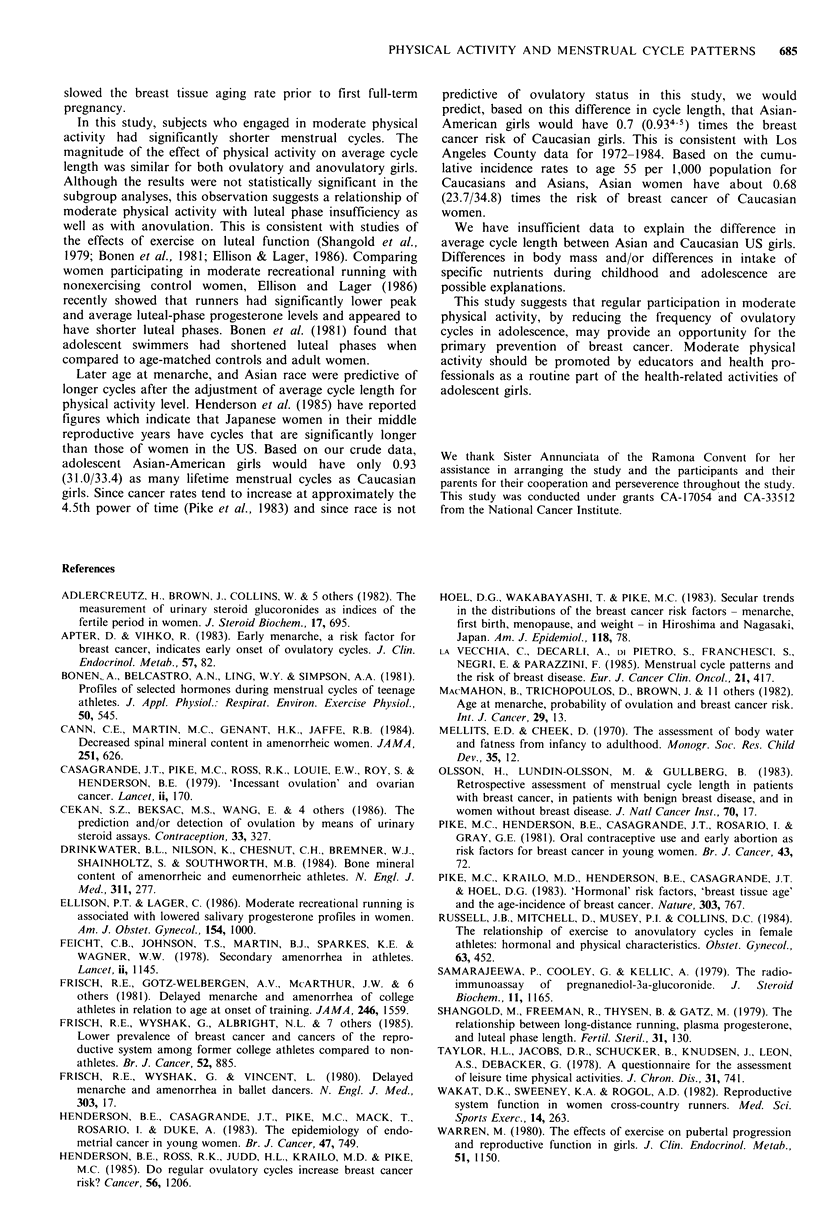# The effects of moderate physical activity on menstrual cycle patterns in adolescence: implications for breast cancer prevention.

**DOI:** 10.1038/bjc.1987.139

**Published:** 1987-06

**Authors:** L. Bernstein, R. K. Ross, R. A. Lobo, R. Hanisch, M. D. Krailo, B. E. Henderson

## Abstract

Girls who engage in strenuous physical activity are often amenorrheic and have recently been reported to be at a reduced risk of breast cancer. To determine whether moderate amounts of exercise affect menstrual cycle patterns and ovulatory frequency in young postmenarcheal girls, the menstrual cycles and physical activity patterns of 168 high school girls were monitored for a 6 month period. Anovulatory cycles were associated with later age at menarche, fewer elapsed years since menarche and greater levels of energy expended per week in physical activity. After adjusting for age at menarche and years since menarche, there was a significant dose-related trend in the risk of anovular menstrual cycles associated with increasing levels of physical activity (1-sided P = 0.03). Major determinants of average cycle length were weekly average energy expenditure (less than or equal to 750 kcal wk-1 associated with cycles that were on average 2.4 days longer), age at menarche (an increase of 0.7 days per year of age) and race (Asians having cycles about 1.9 days longer than Caucasians). Because a major determinant of breast cancer risk may be the cumulative number of ovulatory cycles, these data suggest that regular participation in moderate physical activity, by reducing the frequency of ovulatory cycles in adolescence, may provide an opportunity for the primary prevention of breast cancer.


Br. J. Cancer (1987), 55, 681–685 © The Macmillan Press Ltd., 1987

### The effects of moderate physical activity on menstrual cycle patterns in adolescence: Implications for breast cancer prevention

L. Bernstein, R.K. Ross, R.A. Lobo, R. Hanisch, M.D. Krailo & B.E. Henderson

*Departments of Preventive Medicine and Obstetrics and Gynecology, University of Southern California School of Medicine, Los Angeles, California 90033, USA.*

**Summary** Girls who engage in strenuous physical activity are often amenorrheic and have recently been reported to be at a reduced risk of breast cancer. To determine whether moderate amounts of exercise affect menstrual cycle patterns and ovulatory frequency in young postmenarcheal girls, the menstrual cycles and physical activity patterns of 168 high school girls were monitored for a 6 month period. Anovulatory cycles were associated with later age at menarche, fewer elapsed years since menarche and greater levels of energy expended per week in physical activity. After adjusting for age at menarche and years since menarche, there was a significant dose-related trend in the risk of anovular menstrual cycles associated with increasing levels of physical activity (1-sided $P=0.03$). Major determinants of average cycle length were weekly average energy expenditure ($\leqq 750\,\mathrm{kcal\,wk^{-1}}$ associated with cycles that were on average 2.4 days longer), age at menarche (an increase of 0.7 days per year of age) and race (Asians having cycles about 1.9 days longer than Caucasians). Because a major determinant of breast cancer risk may be the cumulative number of ovulatory cycles, these data suggest that regular participation in moderate physical activity, by reducing the frequency of ovulatory cycles in adolescence, may provide an opportunity for the primary prevention of breast cancer.

Menstrual and ovulatory patterns during adolescence and young adulthood affect the risk of a variety of severe chronic diseases later in life. Age at menarche and cumulative number of ovulatory cycles are considered major determinants of breast cancer risk (Henderson *et al.*, 1985). Young women with menarche before age 12 have a risk of breast cancer that is two times greater than that of women whose age at menarche is 13 or older (Pike *et al.*, 1981). Teenage girls from populations with low breast cancer rates have both later menarche and a lower frequency of ovulatory cycles for a fixed number of years since menarche, than do girls from high risk populations (MacMahon *et al.*, 1982). In particular, Japanese women have significantly lower rates of breast cancer than US women and this is explained, in part, by their later age at menarche and lower body weight (Hoel *et al.*, 1983). In a longitudinal study of young Finnish schoolgirls, Apter and Vihko (1983) showed that girls with early menarche establish ovulatory cycles more quickly than those with later onset of menstruation. Case-control studies have shown that women with breast cancer are more likely than controls to have established a regular menstrual cycle pattern early (Pike *et al.*, 1981; Olsson *et al.*, 1983) and to have maintained a lifelong menstrual cycle pattern that is regular (Olsson *et al.*, 1983; LaVecchia *et al.*, 1985). A woman's risk of ovarian cancer is also directly related to her years of ovulatory menstrual activity (Casagrande *et al.*, 1979); the reverse is true for endometrial cancer (Henderson *et al.*, 1983). Since amenorrheic young women may have reduced bone density (Cann *et al.*, 1984; Drinkwater *et al.*, 1984), their ultimate risk of osteoporosis may be increased. For all of these reasons, factors which influence the onset of menstruation and ovulation and which control the regularity of these events may themselves have important influences on a woman's health.

Premenarcheal girls who engage in strenuous physical activity such as regular ballet dancing, running, or swimming have a considerable delay in the onset of menses (Warren, 1980; Frisch *et al.*, 1981). Secondary amenorrhea may continue throughout the teenage years as long as such strenuous physical activity continues (Feicht *et al.*, 1978; Frisch *et al.*, 1980; Warren, 1980; Frisch *et al.*, 1981; Wakat *et al.*, 1982; Russell *et al.*, 1984). There is now evidence that rigorous physical activity lowers the risk of breast cancer and of reproductive cancers as a whole (Frisch *et al.*, 1985). However, such activity also may increase susceptibility to reduced bone density (Cann *et al.*, 1984; Drinkwater *et al.*, 1984).

Although it is well established that strenuous exercise has profound effects on menstrual activity during adolescence, the effect of moderate physical activity on menstrual cycle and ovulatory patterns is less clear. It has been suggested that moderate exercise may lead to a suppression of luteal function (Shangold *et al.*, 1979; Ellison & Lager, 1986). Thus, moderate physical activity may create a favourable environment in terms of breast (and ovarian) cancer prevention. In the present study, we have monitored high school girls over a six month period to examine the relationship of physical activity patterns, menstrual cycle length and ovulatory frequency.

### Materials and methods

### *Study subjects*

In September, 1984, we invited 276 tenth and eleventh grade students at a Catholic girls' high school, located in Alhambra, California, to participate in the study. The students ranged from 14 to 17 years of age. The study rationale and protocol were explained to all eligible students at an assembly: during the six month study period, all participants would maintain biweekly menstrual cycle calendars and records of physical activity, and would provide early morning, luteal-phase urine samples for two menstrual cycles. At the assembly, students were asked to complete a questionnaire providing information on menstrual cycle history (age at menarche, date of initial menses, usual cycle length, regularity of menses) and relevant background data (date of birth, height and weight). Each girl returning a questionnaire at the end of the assembly was asked to take home a letter of consent which provided details of the study and which was to be signed by one of her parents. All girls who returned consents ($n=174$) were considered participants in the study. None of these girls had ever used oral contraceptives or had ever been pregnant.

### *Data collection procedure*

Biweekly menstrual calendars were collected from partici-

Correspondence: L. Bernstein.
Received 24 October 1986; and in revised form, 20 January 1987.

pants. On the calendars, the girls recorded on a daily basis whether they experienced menstrual bleeding which required a sanitary pad or other protection. They also recorded any weight loss during the two week interval and, for the second of the two weeks, they maintained a daily calendar of their physical activities. The physical activity calendar provided a separate list for recording school-related and non-school related activities. For any activity in which they 'worked up a sweat', subjects documented the type of activity, number of minutes spent in that activity and a relevant measure of the intensity of the activity (e.g., sets of tennis completed or miles run). Menstrual cycles in progress on February 28, 1985 were followed until the next menstrual cycle began. Adequate data (no more than one missing two week calendar segment during the study period) were collected for 168 subjects.

*Sample collection*

Luteal phase urine specimens were collected during two menstrual cycles that were approximately 3 months apart. Each girl provided a first morning urine specimen on cycle day 22 (where day 1 is defined as the first day of menstrual bleeding), and if subsequent menstrual bleeding had not yet begun, a second sample on cycle day 30. All samples were picked up at the girls' homes in the early morning of the day they were collected. They were returned to our laboratory where the total amount of urine collected was measured. Samples were then divided into three 25 ml aliquots and one 2 ml aliquot. These samples were frozen and stored at −20°C for later analysis. We sampled two cycles for 163 girls and one cycle for 5 girls.

*Determination of ovulation*

The concentration of pregnanediol glucoronide in the urine was determined by the method of Samarajeewa *et al.* (1979). Urinary creatinine was measured by the kinetic alkaline picrate method. For each sample the ratio of pregnanediol glucoronide to creatinine (PgC) in $\mu g\,mg^{-1}$ was calculated. Cekan *et al.*, (1986) have demonstrated the utility of this measure in the retrospective assessment of ovulation.

Hormone data from a group of seven ovulatory adult women were obtained to enable us to establish a critical value of PgC for determining whether or not a particular study subject's cycle was ovulatory. These women were participants in a study of the utility of urinary steroid glucoronides to identify the fertile period in women (Adlercreutz *et al.*, 1982). Daily first-morning urine samples had been collected from each of these women for one complete menstrual cycle. The menstrual cycle lengths ranged from 24 to 35 days (mean 29.6). These specimens were assayed concurrently with those of the study subjects. The mean PgC ratio and 95% confidence limits for these control cycles are plotted by the number of days to the onset of the next menstrual cycle in Figure 1. The lower 95% confidence limit for the PgC ratio exceeds $2.0\,\mu g\,mg^{-1}$ for the 11 days prior to the onset of menstrual bleeding. Based on these results, we established $2.0\,\mu g\,mg^{-1}$ as the critical PgC ratio value for judging whether menstrual cycles of our study participants were ovulatory. Thus, if either the day 22 or the day 30 PgC ratio was $>2.0\,\mu g\,mg^{-1}$, we considered the cycle ovulatory. A cycle was anovular if the maximum PgC ratio (for day 22 and, if required, day 30) was $\leq 2.0\,\mu g\,mg^{-1}$ and if the final sample had been collected within 11 days of the onset of the subsequent menstrual cycle. A cycle was indeterminant if the maximum PgC ratio was $\leq 2.0\,\mu g\,mg^{-1}$ and either the final sample has been collected more than 11 days prior to the onset of next menses or the date of onset of the subsequent cycle had not been recorded. Of the 331 cycles assessed in 168 girls, 178 were ovular; 117 were anovular; and 36 were indeterminant.

A summary ovulatory status was assigned to each subject based on her PgC ratio results. If at least one cycle was judged ovular, the subject was designated as ovulatory; if both cycles were adequately assessed and neither was ovular, the subject was considered anovulatory. Adequate data on ovulatory status were available for 146 subjects. Of the 22 participants not assigned a summary ovulatory status, 12 had one anovulatory cycle and one indeterminant cycle (2 with no sample, 3 with no cycle length recorded, and 7 with no sample collected within 11 days of onset of subsequent menses); both sampled cycles were indeterminant for the remaining 10 participants (5 with samples for both cycles collected more than 11 days prior to the onset of subsequent menses, and 5 for whom this was true for one cycle, with no record of cycle length for the other cycle).

**Figure 1** Plot of mean pregnanediol/creatinine ratio ($\mu g\,mg^{-1}$) and 95% confidence limits by numer of days to onset of next menstrual period for the cycles of 7 ovulatory women. Cycle lengths were 24, 27, 29, 30, 30, 31 and 35 days.

*Statistical analysis*

Data on physical activity were summarized (1) by obtaining the average minutes per week that a subject participated in school-related activity and in non-school related activity and (2) by obtaining the kilocalories (kcal) of energy expended in physical activities per week. The energy expenditure for a given activity was based on the product of an intensity code associated with the activity and the total duration of that particular activity in minutes. The intensity codes given by Taylor *et al.* (1978) for various activities were adapted for this study. These codes represent the kcal of energy expended per minute assuming a basal metabolic rate of 60 kcal per hour. The activities (and intensity codes) in which subjects participated were walking (3.5); volleyball and bicycling (4.0); softball (5.0); cheerleading and drill team practice, aerobic and recreational dancing, and exercise class (5.5); gymnastics, swimming, jogging, basketball, and tennis (6.0); and rollerskating (7.0).

Quetelet's index ($100 \times \text{weight}/\text{height}^2$) was used as an index of adiposity. Since this index does not distinguish components of body weight, a second index, the percentage of body fat, was calculated as described by Mellits and Cheek (1970). Percent body fat (BF%) is a function of total body water (TBW):

$$BF\% = 100[1 - TBW/(0.72 \times \text{weight in kg})],$$

where $TBW = -10.313 + 0.252$ (weight in kg) $+ 0.154$ (height in cm). Since the Mellits–Cheek equation was derived using normal, Caucasian, non-physically active girls, the equation likely underestimates lean body mass and overestimates fatness in athletic girls and is not appropriate for use with Asian girls.

Analysis of variance methods were used to evaluate the within subject and among subjects variation in cycle length. The average cycle length over the study period was calculated for each subject and multiple linear regression methods were used to establish factors associated with this measure.

Because a number of variables had skewed distributions, the Kruskal–Wallis test was used to evaluate differences in means between ovular and anovular subjects and between Asian and Caucasian subjects. Multiple logistic regression methods were used to assess the magnitude of effects of factors associated with anovular status. For the analysis of physical activity, quintiles of average weekly energy expenditure were examined to determine whether there was an increasing trend in the likelihood of anovulation with increasing level of physical activity.

### Results

Over the study period, the 168 subjects documented 643 complete menstrual cycles (3.8 per subject). Cycles ranged in length from 10 to 90 days, and the average cycle length was 30.3 days (standard deviation 7.8 days). Ninety-one percent of the cycles were between 20 and 40 days in length. An analysis of variance, which was used to evaluate sources of variation, revealed significantly more variation in cycle length between subjects than within the cycles recorded for any one subject ($P<0.0001$).

### *Ovulatory status*

Ovulatory status was not determined for 22 girls (13.1%). Subjects for whom we had sufficient information to determine ovulatory status are compared to those with undetermined status in Table I. The subjects with undetermined status were similar to those we considered anovular in terms of age at menarche, years since menarche and indices of physical activity (total minutes and kcal per week). However, they had longer average cycle lengths than both groups, as might be expected from the criteria used to assess whether adequate sampling had occurred. All three groups were similar in terms of body size measures.

Thirty of the 146 subjects with determined ovulatory status (20.5%) were anovular. Although there were no differences in age between anovular and ovular girls, those who were anovular began menstruating at a significantly later age and had menstruated significantly fewer years than those who were ovular (Table I). There was also a significant difference in average cycle length with anovular subjects reporting cycles that were on average 4 days shorter than those of girls who were ovulatory. Anovular girls spent greater amounts of time in both school-related and non-school related physical activity, consistently expending more energy (kcal) per week in exercise over the six month observation period. The two groups did not differ on measures of body size and adiposity.

The proportions of subjects with anovulatory status did not differ by race. Six of 26 Asian subjects (23.1%) were anovulatory compared with 24 of 120 Caucasian subjects (20.0%).

Table II shows the distribution of subjects by age at menarche and gynaecologic age (number of months since onset of menses) according to ovulatory status. The percentage of subjects with anovular status decreases with increasing gynaecologic age from 37% in the first twenty-

**Table I** Characteristics of study population by ovulatory status (Mean ± s.d. presented)

| Characteristic | *Status* Not assigned N=22 | Anovulatory N=30 | Ovulatory N=116 | 2-sided P-value[a] |
|---|---|---|---|---|
| Age | 16.0±0.8 | 16.2±0.9 | 16.3±0.9 | 0.61 |
| Menses | | | | |
| Age at menarche | 12.5±1.2 | 12.5±1.0 | 11.7±1.2 | <0.001 |
| Gynaecologic age | 3.3±1.6 | 3.4±1.4 | 4.4±1.4 | <0.001 |
| Average cycle length (days) | 38.8±7.6 | 27.1±3.6 | 31.3±6.2 | <0.001 |
| Body size | | | | |
| Height (cm) | 161.1±5.8 | 163.0±6.0 | 160.9±5.8 | 0.06 |
| Weight (kg) | 55.7±14.5 | 56.3±8.8 | 54.2±7.9 | 0.22 |
| Quetelet's index | 2.14±0.50 | 2.12±0.29 | 2.09±0.24 | 0.93 |
| Body fat (%)[b] | 31.0±5.8 | 27.6±4.8 | 27.6±4.3 | 0.75 |
| Physical activity | | | | |
| School-related (min/week) | 64.1±103.2 | 89.0±132.0 | 71.4±120.3 | 0.14 |
| Non-school related (min/week) | 112.5±139.9 | 96.0±85.4 | 89.6±85.3 | 0.71 |
| Energy expenditure (kcal/week) | 1037±1144 | 1118±938 | 952±848 | 0.10 |

[a]Kruskal–Wallis test, comparing anovulatory and ovulatory subjects; [b]Based on N=14, N=24 and N=94 Caucasian girls respectively.

**Table II** Distribution of subjects by ovulatory status, age at menarche and gynaecologic age

| Gynaecologic age (mos) | *Age at menarche* ≤11 Anovular | ≤11 Ovular | 12 Anovular | 12 Ovular | ≥13 Anovular | ≥13 Ovular | Total Anovular | Total Ovular | Total % Anovular |
|---|---|---|---|---|---|---|---|---|---|
| ≤24 | – | – | 2 | 5 | 9 | 14 | 11 | 19 | 36.7 |
| 25–36 | 2 | 4 | 1 | 11 | 8 | 9 | 11 | 24 | 31.4 |
| 37–48 | 1 | 9 | 3 | 16 | 0 | 8 | 4 | 33 | 10.8 |
| 49+ | 1 | 34 | 3 | 5 | 0 | 1 | 4 | 40 | 9.1 |
| Total | 4 | 47 | 9 | 37 | 17 | 32 | 30 | 116 | 20.5 |
| % Anovular | 7.8 | | 19.6 | | 34.7 | | 20.5 | | |

four months to 9% at 60 or more months (test for trend, 1-sided $P<0.001$). The percentage of anovular cycles also increases with increasing age at menarche from about 8% for girls with menarche at age 11 or younger to nearly 35% for girls with menarche at age 13 or older (test for trend, 1-sided $P=0.001$). Age at menarche and gynaecologic age are highly correlated since girls who were studied at relatively short intervals after menarche tended to be those with late menarche. Using logistic regression analysis to mutually adjust each factor for the other, the magnitude of each effect is reduced and neither factor is statistically significant after adjustment for the other. When gynaecologic age and age at menarche are fitted jointly, the regression slope for gynaecologic age is $-2.8\%$ per year (1-sided $P=0.11$) and that for age at menarche is 4.3% per year (1-sided $P=0.07$). Thus, it is difficult to disentangle the effects of each factor on the probability of anovulation.

The data for average weekly energy expenditure were categorized into quintiles to evaluate the relationship between anovulatory status and physical activity. As shown in Table III, there was a significant trend in the risk of anovulation with increasing average weekly energy expenditure (1-sided $P=0.03$) after adjustment for gynaecologic age and age at menarche. Girls who engaged in moderate physical activity (averaging more than 600 kcal of energy expenditure per week) were 2.9 times more likely to be anovular than were girls who engaged in lesser amounts of physical activity (2-sided $P=0.03$). There was no association between either measure of adiposity and ovulatory status.

**Table III** Relative risk (RR) of anovulatory menstrual cycles

| | *Average weekly energy expenditure (kcal)* | | | | |
|---|---|---|---|---|---|
| *Status* | *≦300* | *301–600* | *601–930* | *931–1400* | *1401+* |
| Anovulatory | 4 | 3 | 6 | 10 | 7 |
| Ovulatory | 26 | 28 | 23 | 18 | 24 |
| RR[a] | 1.00 | 0.91 | 2.33 | 3.85 | 2.33 |

[a]Adjusted for gynaecologic age and age at menarche.

*Cycle length*

Analyses of cycle length were conducted by summarizing each subject's cycle length record by her average cycle length. Energy expenditure was dichotomized at the median ($\leq 750\,\text{kcal wk}^{-1}$ *vs.* $>750\,\text{kcal wk}^{-1}$). Based on a stepwise multiple linear regression analysis, predictors of average cycle length (and 2-sided $P$-values conditional on other variables in the model) were, in order of selection, average weekly energy expenditure ($P=0.01$), age at menarche ($P=0.08$), and race (Asian *vs.* Caucasian; $P=0.11$). In the final model, expending 750 kcal or less of energy per week in physical activity was associated with cycles that were on average 2.4 days longer than those of more physically active girls. Average cycle length increased about 0.7 days per year of age at menarche and was, on average, 1.9 days longer for Asians than for Caucasians.

We examined further the relationship of moderate physical activity, age at menarche and average cycle length by ovulatory status. The effect of physical activity was strongest for girls with undetermined ovulatory status. In this subgroup, girls who expended more than 750 kcal of energy per week in physical activity had cycle lengths that were, on average, 4.1 days shorter than those of less active girls (2-sided $P=0.27$); age at menarche had little effect with average cycle length increasing 0.3 days per year of age at menarche (2-sided $P=0.8$). Results for anovulatory and ovulatory girls were similar. For anovulatory girls, cycle length increased, on average, 1.1 days per year of age at menarche (2-sided $P=0.07$) and was, on average, 1.4 days shorter for girls expending more than 750 kcal energy per week in physical activity. In ovulatory girls, average cycle length increased, on average, 0.9 days per year of age at menarche (2-sided $P=0.07$) and was, on average, 1.2 days shorter for girls expending more than 750 kcal energy per week in physical activity.

**Table IV** Comparison of characteristics by race (Mean ± s.d. presented.)

| *Characteristic* | *Asian N=36* | *Caucasian N=132* | *2-sided P-value*[a] |
|---|---|---|---|
| Age | 16.2±1.0 | 16.3±0.8 | 0.90 |
| Menses | | | |
| Age at menarche | 12.2±1.2 | 11.9±1.2 | 0.27 |
| Gynaecologic age | 3.9±1.4 | 4.2±1.6 | 0.57 |
| Average cycle length (days) | 33.4±7.5 | 31.0±6.6 | 0.05 |
| Body size | | | |
| Height (cm) | 158.7±5.2 | 162.0±5.9 | 0.004 |
| Weight (kg) | 51.2±6.8 | 55.8±9.5 | 0.004 |
| Quetelet's index | 2.03±0.22 | 2.12±0.31 | 0.10 |
| Body fat (%) | – | 27.9±4.7 | – |
| Physical activity | | | |
| School-related (min/week) | 75.3±140.3 | 73.2±114.3 | 0.93 |
| Non-school related (min/week) | 76.6±95.4 | 98.5±93.1 | 0.05 |
| Energy expenditure (kcal/week) | 939±1010 | 1023±878 | 0.12 |

[a]Kruskal–Wallis test, comparing Asians and Caucasians.

Characteristics of menses, body size and physical activity are compared in Table IV for Asians and Caucasians. In addition to having significantly longer menstrual cycles, Asians were both significantly shorter in stature and lighter in weight. There was no difference in age between the two groups of girls, nor did they differ on gynaecologic age. Although both groups participated equally in school-related physical activity, Caucasian girls spent significantly more time participating in non-school related physical activities, averaging 22 minutes more per week than Asian girls.

### Discussion

The effects of age at menarche and gynaecologic age on the probability of anovulation in our study group are consistent with those reported by others (MacMahon *et al.*, 1982; Apter & Vihko, 1985). Girls who participated in moderate physical activity, averaging more than 600 kcal of energy expenditure in such activity per week, were significantly more likely than less active girls to be classified as anovular. Such moderate physical activity represents sustained participation of two or more hours per week in activities like aerobic exercise classes, swimming, jogging or tennis.

In a recent publication, Henderson *et al.* (1985) summarized the evidence suggesting that breast cancer risk is directly related to the cumulative number of ovulatory cycles. Any factors which modify the age at onset of menstruation, frequency of ovulation or cycle length pattern could markedly reduce a woman's lifetime risk of developing breast [as well as ovarian (Casagrande *et al.*, 1979)] cancer. The results of this study suggest that participation in sustained levels of moderate physical activity during adolescence may alter breast cancer risk by reducing the frequency of ovulatory cycles. The results of a recent study of female college alumnae show that former college athletes have about half the breast cancer risk of non-athletes (Frisch *et al.*, 1985). This reduction in risk is consistent with that which would be predicted using the model of breast tissue aging proposed by Pike *et al.* (1983) if the increased frequency of anovular cycles resulting from exercise during adolescence slowed the breast tissue aging rate prior to first full-term pregnancy.

In this study, subjects who engaged in moderate physical activity had significantly shorter menstrual cycles. The magnitude of the effect of physical activity on average cycle length was similar for both ovulatory and anovulatory girls. Although the results were not statistically significant in the subgroup analyses, this observation suggests a relationship of moderate physical activity with luteal phase insufficiency as well as with anovulation. This is consistent with studies of the effects of exercise on luteal function (Shangold *et al.*, 1979; Bonen *et al.*, 1981; Ellison & Lager, 1986). Comparing women participating in moderate recreational running with nonexercising control women, Ellison and Lager (1986) recently showed that runners had significantly lower peak and average luteal-phase progesterone levels and appeared to have shorter luteal phases. Bonen *et al.* (1981) found that adolescent swimmers had shortened luteal phases when compared to age-matched controls and adult women.

Later age at menarche, and Asian race were predictive of longer cycles after the adjustment of average cycle length for physical activity level. Henderson *et al.* (1985) have reported figures which indicate that Japanese women in their middle reproductive years have cycles that are significantly longer than those of women in the US. Based on our crude data, adolescent Asian-American girls would have only 0.93 (31.0/33.4) as many lifetime menstrual cycles as Caucasian girls. Since cancer rates tend to increase at approximately the 4.5th power of time (Pike *et al.*, 1983) and since race is not predictive of ovulatory status in this study, we would predict, based on this difference in cycle length, that Asian-American girls would have 0.7 ($0.93^{4.5}$) times the breast cancer risk of Caucasian girls. This is consistent with Los Angeles County data for 1972–1984. Based on the cumulative incidence rates to age 55 per 1,000 population for Caucasians and Asians, Asian women have about 0.68 (23.7/34.8) times the risk of breast cancer of Caucasian women.

We have insufficient data to explain the difference in average cycle length between Asian and Caucasian US girls. Differences in body mass and/or differences in intake of specific nutrients during childhood and adolescence are possible explanations.

This study suggests that regular participation in moderate physical activity, by reducing the frequency of ovulatory cycles in adolescence, may provide an opportunity for the primary prevention of breast cancer. Moderate physical activity should be promoted by educators and health professionals as a routine part of the health-related activities of adolescent girls.

We thank Sister Annunciata of the Ramona Convent for her assistance in arranging the study and the participants and their parents for their cooperation and perseverence throughout the study. This study was conducted under grants CA-17054 and CA-33512 from the National Cancer Institute.

### References

ADLERCREUTZ, H., BROWN, J., COLLINS, W. & 5 others (1982). The measurement of urinary steroid glucoronides as indices of the fertile period in women. *J. Steroid Biochem.*, **17**, 695.

APTER, D. & VIHKO, R. (1983). Early menarche, a risk factor for breast cancer, indicates early onset of ovulatory cycles. *J. Clin. Endocrinol. Metab.*, **57**, 82.

BONEN, A., BELCASTRO, A.N., LING, W.Y. & SIMPSON, A.A. (1981). Profiles of selected hormones during menstrual cycles of teenage athletes. *J. Appl. Physiol.: Respirat. Environ. Exercise Physiol.*, **50**, 545.

CANN, C.E., MARTIN, M.C., GENANT, H.K., JAFFE, R.B. (1984). Decreased spinal mineral content in amenorrheic women. *JAMA*, **251**, 626.

CASAGRANDE, J.T., PIKE, M.C., ROSS, R.K., LOUIE, E.W., ROY, S. & HENDERSON, B.E. (1979). 'Incessant ovulation' and ovarian cancer. *Lancet*, **ii**, 170.

CEKAN, S.Z., BEKSAC, M.S., WANG, E. & 4 others (1986). The prediction and/or detection of ovulation by means of urinary steroid assays. *Contraception*, **33**, 327.

DRINKWATER, B.L., NILSON, K., CHESNUT, C.H., BREMNER, W.J., SHAINHOLTZ, S. & SOUTHWORTH, M.B. (1984). Bone mineral content of amenorrheic and eumenorrheic athletes. *N. Engl. J. Med.*, **311**, 277.

ELLISON, P.T. & LAGER, C. (1986). Moderate recreational running is associated with lowered salivary progesterone profiles in women. *Am. J. Obstet. Gynecol.*, **154**, 1000.

FEICHT, C.B., JOHNSON, T.S., MARTIN, B.J., SPARKES, K.E. & WAGNER, W.W. (1978). Secondary amenorrhea in athletes. *Lancet*, **ii**, 1145.

FRISCH, R.E., GOTZ-WELBERGEN, A.V., MCARTHUR, J.W. & 6 others (1981). Delayed menarche and amenorrhea of college athletes in relation to age at onset of training. *JAMA*, **246**, 1559.

FRISCH, R.E., WYSHAK, G., ALBRIGHT, N.L. & 7 others (1985). Lower prevalence of breast cancer and cancers of the reproductive system among former college athletes compared to non-athletes. *Br. J. Cancer*, **52**, 885.

FRISCH, R.E., WYSHAK, G. & VINCENT, L. (1980). Delayed menarche and amenorrhea in ballet dancers. *N. Engl. J. Med.*, **303**, 17.

HENDERSON, B.E., CASAGRANDE, J.T., PIKE, M.C., MACK, T., ROSARIO, I. & DUKE, A. (1983). The epidemiology of endometrial cancer in young women. *Br. J. Cancer*, **47**, 749.

HENDERSON, B.E., ROSS, R.K., JUDD, H.L., KRAILO, M.D. & PIKE, M.C. (1985). Do regular ovulatory cycles increase breast cancer risk? *Cancer*, **56**, 1206.

HOEL, D.G., WAKABAYASHI, T. & PIKE, M.C. (1983). Secular trends in the distributions of the breast cancer risk factors – menarche, first birth, menopause, and weight – in Hiroshima and Nagasaki, Japan. *Am. J. Epidemiol.*, **118**, 78.

LA VECCHIA, C., DECARLI, A., DI PIETRO, S., FRANCHESCI, S., NEGRI, E. & PARAZZINI, F. (1985). Menstrual cycle patterns and the risk of breast disease. *Eur. J. Cancer Clin. Oncol.*, **21**, 417.

MACMAHON, B., TRICHOPOULOS, D., BROWN, J. & 11 others (1982). Age at menarche, probability of ovulation and breast cancer risk. *Int. J. Cancer*, **29**, 13.

MELLITS, E.D. & CHEEK, D. (1970). The assessment of body water and fatness from infancy to adulthood. *Monogr. Soc. Res. Child Dev.*, **35**, 12.

OLSSON, H., LUNDIN-OLSSON, M. & GULLBERG, B. (1983). Retrospective assessment of menstrual cycle length in patients with breast cancer, in patients with benign breast disease, and in women without breast disease. *J. Natl Cancer Inst.*, **70**, 17.

PIKE, M.C., HENDERSON, B.E., CASAGRANDE, J.T., ROSARIO, I. & GRAY, G.E. (1981). Oral contraceptive use and early abortion as risk factors for breast cancer in young women. *Br. J. Cancer*, **43**, 72.

PIKE, M.C., KRAILO, M.D., HENDERSON, B.E., CASAGRANDE, J.T. & HOEL, D.G. (1983). 'Hormonal' risk factors, 'breast tissue age' and the age-incidence of breast cancer. *Nature*, **303**, 767.

RUSSELL, J.B., MITCHELL, D., MUSEY, P.I. & COLLINS, D.C. (1984). The relationship of exercise to anovulatory cycles in female athletes: hormonal and physical characteristics. *Obstet. Gynecol.*, **63**, 452.

SAMARAJEEWA, P., COOLEY, G. & KELLIC, A. (1979). The radioimmunoassay of pregnanediol-3a-glucoronide. *J. Steroid Biochem.*, **11**, 1165.

SHANGOLD, M., FREEMAN, R., THYSEN, B. & GATZ, M. (1979). The relationship between long-distance running, plasma progesterone, and luteal phase length. *Fertil. Steril.*, **31**, 130.

TAYLOR, H.L., JACOBS, D.R., SCHUCKER, B., KNUDSEN, J., LEON, A.S., DEBACKER, G. (1978). A questionnaire for the assessment of leisure time physical activities. *J. Chron. Dis.*, **31**, 741.

WAKAT, D.K., SWEENEY, K.A. & ROGOL, A.D. (1982). Reproductive system function in women cross-country runners. *Med. Sci. Sports Exerc.*, **14**, 263.

WARREN, M. (1980). The effects of exercise on pubertal progression and reproductive function in girls. *J. Clin. Endocrinol. Metab.*, **51**, 1150.